# Observations on Dental Caries

**Published:** 1891-09

**Authors:** A. M. Ross

**Affiliations:** Springfield, Mass.


### OBSERVATIONS ON DENTAL CARIES.

BY DR A. M. ROSS, SPRINGFIELD, MASS.

THE practical mai is never satisfied with any theory that cannot be reconciled with all the facts that are known in relation to the subject, and he is disposed to at least a kindly criticism of a still prevalent custom of ignoring facts not compatible with a theory, or the twisting of other facts to conform to it.

Dr. Thomas Fillebrown has proclaimed a broad truth in saying recently that a bushel of disease germs disposed on healthy tissue can produce no ill effect. He means, of course, where the resistive power is sufficient to inhibit them or digest them. They must have suitable soil for development, and that varies in different cases. Seed-corn from the same ear may bear a far better fruitage in one garden than in another. Self-evident similes would be useless with the difference that exists between pathogenic and saprophytic microbes, if it were not that animal and vegetable organisms are subjected to the effects of both, and that there is as much difference between them as there is between the thieves and gamblers of a community and the honest scavengers at work in our streets and alley-ways. Careful study of the subject of dental caries proves beyond a doubt that the disintegration of the structure is caused by organisms. But that they are the prime cause of caries is far from proven.

The waste product of these organisms is not destructive in some cases. The "miniature acid factory" is often quite well established in teeth, and a return of vigorous resistive power puts a stop to operations, and it doesn't matter whether this arresting of caries is permanent or not, the fact itself disproves a theory of waste products that alone are sufficient to destroy the tooth. But the fact is, it is often a permanent arresting of decay. There is a curious fact in connection with this subject that I do not remember having seen mentioned by Dr. Miller or others. It is found that the slower or the nearer to an arrested state of decay the nearer to the initial point of breaking down of the lime-salts are the micro-organisms. And that in rapid colorless caries and in senile decay the field beyond the closest approach of the organisms is very large in which not a single canaliculus is distended. Possibly the reverse of this would be more compatible with a theory of waste-products. I leave it for those better qualified than myself to say.

If we read aright the conclusions of bacteriologists we find that the conditions favorable to the saprophytes are not dependent upon external conditions. Either death of the tissue or its lack of resistive force—very nearly an equivalent—is necessary for the development of micro-organisms, etc. The highest expression of vitality is sufficient to prevent or to arrest existing caries of the teeth. Another question presents itself that arises from two important considerations,—the first, it may be said, is based on the fact that the word stasis has no application in life, and the other consideration has its basis upon a certain microscopical fact. Movement is everywhere. In all systems of life it is a gradient or retrogradient continually. We cannot except any part of a system to this law. Ground sections of carious teeth treated with one-eighth per cent. good chloride show the primary changes in the dentine to be beyond the point of softening at all.

The question that arises, then, is this: Is the degree of non-resistance a negative state in living teeth, or is it one of irritability, denoting a retrograde movement that of itself is destructive of tissue? If we take long broad views of the subject our ideas regarding these micro-organisms may change as we see the necessity of them in the economy of nature. We may discover that dental caries is the result of impaired resistive forces, or more properly impaired vitality. Millions of these very organisms constantly present in the mouth, notwithstanding the utmost care taken of it, force us to believe that their inhibition by artificial means can have only temporary effects: the destructive waste-products can be successfully met alone by a continuous antagonism from an adequate energy.

We have found that combinations of gold and tin, gold and amalgam, etc., have had a more preserving effect in some cases than any of the metals or alloys used singly. In some other cases it has made little or no difference. Are we justified in concluding that the favorable results are due to a destruction of acid-producers about the teeth, or, perhaps more reasonably, to conclude from all the evidence that by such combinations of material resistive forces are stimulated to greater activity?

